# The Complete Genome Sequence of the San Miguel Sea Lion Virus-8 Reveals that It Is Not a Member of the Vesicular Exanthema of Swine Virus/San Miguel Sea Lion Virus Species of the *Caliciviridae*

**DOI:** 10.1128/genomeA.01286-14

**Published:** 2014-12-11

**Authors:** John D. Neill

**Affiliations:** Ruminant Diseases and Immunology Research Unit, National Animal Disease Center, Department of Agriculture, ARS, Ames, Iowa, USA

## Abstract

The complete genome sequence of the San Miguel sea lion virus-8 (SMSV-8) was determined in this study. A comparison of this sequence to other calicivirus sequences in GenBank showed that this virus is genetically distinct from the vesicular exanthema of swine virus/San Miguel sea lion virus (VESV/SMSV) strains and belongs to a novel clade within the *Vesivirus* genus.

## GENOME ANNOUNCEMENT

The *Caliciviridae* is composed of five recognized genera, *Norovirus*, *Sapovirus*, *Lagovirus*, *Vesivirus*, and *Nebovirus*. The *Vesivirus* genus contains viruses that primarily infect animals only, such as feline calicivirus, vesicular exanthema of swine virus (VESV), San Miguel sea lion virus (SMSV), and viruses that have not been assigned to a specific group. The first isolated SMSV type, SMSV-1, was reported in 1972 from vesicular lesions on the flipper of a California sea lion (1). The virus was shown to have biophysical properties in common with vesicular exanthema of swine viruses (2). The isolation of SMSV-8 was reported in 1981 from vesicular lesions on the flippers of northern fur seals and possessed the typical calicivirus morphology (3).

SMSV-8 was propagated on Vero cells and supernatant stocks stored at -80°C. To prepare for sequencing, viral RNA was purified following the treatment of the virus stock with a nuclease cocktail (4). The genomic RNA was sequenced using a random-primed single-primer method for the synthesis of barcoded double-stranded cDNA (5). Briefly, a single-tube reverse transcriptase followed by two second-strand cDNA synthesis reactions was performed using 20-mer primers of known sequence with 8 random nucleotides on the 3′ ends. The cDNA was amplified using primers with the same 20-mer sequence. The amplified cDNA was prepared for sequencing using the Ion Torrent sequencing platform (Life Technologies, Inc., Grand Island, NY). The viral genome was assembled with SeqMan NGen software (DNAStar, Inc., Madison, WI) using *de novo* assembly of sequences and alignment to mink calicivirus strain MCV-DL/2007/CN (GenBank accession no. JX847605). The 3′ terminal sequence was confirmed by 3′ RACE.

The SMSV-8 library yielded 36,783 (34,978 virus) sequences. This number of sequences provided an average coverage over the genome of 489×. The genome length of SMSV-8 is 8,477 bases, excluding the poly(A) tail. The genomic RNA contains 3 open reading frames (ORFs), the first encoding the nonstructural polyprotein, the second encoding the capsid protein precursor (VP-1), and the third encoding a small basic protein (VP-2). A phylogenetic analysis of the genome sequence showed that the most closely related caliciviruses to SMSV-8 are members of the *Vesivirus* genus. The virus with the greatest genetic relatedness to SMSV-8 is mink calicivirus (MCV) (GenBank accession no. JX847605), at 71% identity. When the amino acid sequences of the three ORFs were compared to those of other vesiviruses, SMSV-8 was found to be distantly related. The amino acid sequences of ORF1, ORF2, and ORF3 have 73%, 63%, and 48% identity, respectively, with those of MCV. With respect to other caliciviruses, SMSV-8 has 55%, 39%, and 34% identity with the three ORFs of SMSV-1 (Genbank accession no. U15301), 55%, 38%, and 35% identity to VESV A48 (Genbank accession no. U76874), and 61%, 46%, and 48% identity to calicivirus Allston 2008/US (Genbank accession no. GQ475302). These data indicate that SMSV-8 is a novel member of the *Vesivirus* genus. This supports Seal et al. (6) and Reid et al. (7), who suggested that SMSV-8 belongs to a separate calicivirus group based on a lack of reactivity using immunologic and genetic screening methods.

### Nucleotide sequence accession number.

The genomic sequence of San Miguel sea lion virus-8 has been deposited in GenBank with the accession no. KM244552.
